# The War Charities Committee

**Published:** 1916-07-22

**Authors:** 


### The Hospital

The Workers' Newspaper of Administrative Medicine and Institutional Life, Administration, National Insurance and Health.

No. 1572, Vol. LX. SATURDAY, JULY 22, 1916. PRICE ONE PENNY.

### THE WAR CHARITIES COMMITTEE.

So long ago as August 28, 1915, we urged in these columns the need for the application, to the various charitable schemes taking origin in connection with the war, of business methods and sound financial principles. A long experience of charitable enterprises had taught us that people possessed of excellent motives and good intentions are not always alive in a full measure to the responsibility involved in dealing with money for which they have undertaken to act as trustees. To not a few, vouchers and accounts and balance-sheets seem unnecessary when philanthropic aspiration is the end to be pursued. In addition, the situation created by the war carried obvious opportunities for trickery and fraud. Even a great national crisis does not repress in every individual a perception of the chance of exploiting for personal ends the patriotic promptings of the general public. In short, the creation and multiplication of funds having as a nominal purpose some object which touched widespread sympathies carried risks of mismanagement, of blundering, and of inefficiency; and, on quite another plane, it gave an opening to the ingenious individual whose aim is personal profit disguised as patriotism or philanthropy. Even the well-meaning need business supervision, while for the others exposure is the only remedy; unfortunately it is not always a successful remedy. Such were the considerations which led us in quite early days to call for full and verified statements of accounts from all committees charged with war charities. There were large public reasons for this demand. The organisations were numerous and the country was in no mood for a cold analysis of their several claims and merits. Money was being given freely under the desire to be helpful wherever help was declared to be necessary. Nothing was easier than to organise a committee and to adorn it with the names of decorative persons whose alphabetical distinctions appear to the unwary to be guarantees of efficiency. In other words, there was a plain risk of waste from improper or incapable management, and from overlapping, not to speak of grosser dangers. These things, bad in themselves, meant the possibility of other mischiefs, for the blundering or incapacity of a few would, when discovered, be apt to throw discredit even on more seemly enterprises; and, with the prospect of a long struggle and heavy financial demands in front of us, the need was less for a short-lived outburst of enthusiasm than for a steady, sustained, and ordered purpose. To this end, accurately kept accounts and verified balance-sheets form a not unimportant contribution, and we therefore urged publication of such documents by all the committees.

The Report of the Committee on War Charities which was issued a few days ago contains an abundant justification of the claim we ventured to state; indeed it reveals a position which demands the most urgent attention of all who desire that the generous intentions of the public shall not be defeated either by incapacity on the one hand or by fraud on the other. If legislation is necessary, and this is more than probable, our legislators must find opportunity to achieve it. At present it seems that any individual may collect funds for any purpose which pleases him. He may dignify his proposal with any title which strikes his fancy, and may multiply "patrons" and "vice-presidents" galore. Yet there is no obligation on him to keep a proper record of his receipts and expenditure, and, short of actual and open fraud, he may make a comfortable living out of the business and may live and flourish in the odour of sanctity. The same laxity of control, of course, extends to enterprises of undoubted good faith, and there is equal evidence that the results, in spite of the excellent motives of the promoters, have been very different from those intended by the good people who have found the money. Such things have long been known to persons in touch with the charitable and philanthropic world, but the war has extended and emphasised them. This plainly appears in the Report now before us, and while its lessons have a pressing immediate application, it is none the less true that they are of permanent significance. War charities have, in all probability, still a long innings before them, and much damage will remain for repair long after the fighting has ceased. Peace, too, will add problems of its own. It is therefore important that without delay the money contributed by a generous public shall be used both wisely and well, and that those who undertake its distribution shall be led, gently or otherwise, to see the necessity of ordering their activities on a sound business basis.

A few of the experiences recited in the Committee's Report will enforce this contention. In one instance the whole of the money collected was spent in "working expenses," and the fund actually shows an adverse balance, though the committee consisted of some eighty ladies and earls and members of Parliament appeared as vice-presidents. In a second case, out of a total of £129,000 there is a balance of £42,000 which has not been traced. And in a third example a sum of £260 was collected in four months, and of this only £14 was spent in charitable purposes, in spite of the appearance of a number of "titled persons" on the list of patrons. Committees have been found to be merely "nominal," and there has been a reckless readiness on the part of many to lend their names to schemes over which they exercised no control. The obvious remedy for such evils is to make it illegal to appeal for money for philanthropic or charitable purposes unless the enterprise is formally registered with some responsible authority. Registration must demand certain conditions, such, for example, as the existence of a properly constituted committee, the establishment of a separate and independent banking account, the accurate record of receipts and expenditure, and the publication of a duly audited statement of accounts. No compulsion short of the law can be effective in these directions; and, while the need for this compulsion is made manifest by recent experiences, it is really applicable over a wide field if money intended for worthy ends is to be efficiently managed and protected from incapacity and fraud.

